# Modified Gexia-Zhuyu Tang inhibits gastric cancer progression by restoring gut microbiota and regulating pyroptosis

**DOI:** 10.1186/s12935-024-03215-6

**Published:** 2024-01-09

**Authors:** Tingting Zhao, Zhijian Yu

**Affiliations:** 1 Shanghai Key Laboratory of Veterinary Biotechnology, School of Agriculture and Biology, Shanghai Jiao Tong University, No. 800, Dongchuan Road, Shanghai City, 200240 China; 2 School of Traditional Chinese Medicine, Southern Medical University,Third Level Research Laboratory of State Administration of Traditional Chinese Medicine, Guangdong Provincial Key Laboratory of Chinese Medicine Pharmaceutics, Guangdong Provincial Engineering Laboratory of Chinese Medicine Preparation Technology, No. 1023-1063, Shatai South Road, Guangzhou City, 510515 Guangdong Province China

**Keywords:** GC, Modified GZT, Intestinal flora, Pyroptosis

## Abstract

**Background:**

Gexia-Zhuyu Tang (GZT), a traditional Chinese medicine formula, is used to treat a variety of diseases. However, its roles in gastric cancer (GC) remain unclear.

**Objective:**

The aim of this study was to explore the roles and underlying molecular mechanisms of modified GZT in GC.

**Methods:**

The effects of modified GZT on GC were investigated by constructing mouse xenograft models with MFC cell line. The fecal samples from low-dose, high-dose, and without modified GZT treatment groups were collected for the 16S rRNA gene sequencing and fecal microbiota transplantation (FMT). Histopathological alterations of mice were evaluated using the hematoxylin–eosin (HE). Immunohistochemical (IHC) analysis with Ki67 and GSDMD was performed to measure tissue cell proliferation and pyroptosis, respectively. Proteins associated with pyroptosis, invasion, and metastasis were detected by Western blotting. Enzyme-linked immunosorbent assay (ELISA) was used to assess inflammation-related factors levels.

**Results:**

Modified GZT inhibited GC tumor growth and reduced metastasis and invasion-related proteins expression levels, including CD147, VEGF, and MMP-9. Furthermore, it notably promoted caspase-1-dependent pyroptosis, as evidenced by a dose-dependent increase in TNF-α, IL-1β, IL-18, and LDH levels, along with elevated protein expression of NLRP3, ASC, and caspase-1. Additionally, modified GZT increased species abundance and diversity of the intestinal flora. FMT assay identified that modified GZT inhibited GC tumor progression through regulation of intestinal flora.

**Conclusions:**

Modified GZT treatment may promote pyroptosis by modulating gut microbiota in GC. This study identifies a new potential approach for the GC clinical treatment.

**Supplementary Information:**

The online version contains supplementary material available at 10.1186/s12935-024-03215-6.

## Background

Gastric cancer (GC) is a prevalent malignancy, ranking fifth in terms of new cases in 2020 and fourth in terms of mortality, causing approximately 800,000 deaths [1, 2]. Even with various treatment options like surgery, chemotherapy, radiotherapy, drugs, and immunotherapy, the prognosis for patients with GC remains unsatisfactory [3]. Therefore, the identification of alternative and effective treatments for GC are of paramount importance.

Having been practiced for over 5000 years, Traditional Chinese Medicine (TCM) has gained recognition as a credible and promising alternative therapy for cancer. Gexia-Zhuyu Tang (GZT) is a TCM Formula (TCMF) that shows promise in treating chronic diseases [4]. It is composed of 12 Chinese herbal medicines, including *Peach* ker*nel*, *Safflower*, *Angelica sinensis*, *Chuanxiong*, *Red peony*, *Peony bark*, *YanhuSuo*, *Wulingzhi*, *Wuyao*, *Fructus Aurantii*, *Xiangfu*, and *Licorice*.Previous studies have identified the paeoniflorin and glycyrrhizin components in the GZT mixture through High-performance liquid chromatography (HPLC) analysis, which have the inhibitory effects on GC development [4–8]. In addition, several herbs in GZT have anti-GC tumor effects, such as *Safflower*, *Chuanxiong*, and *Angelica sinensis* [9–11]. However, the roles and potential molecular mechanisms of GZT in GC remain unclear. In this study, we have modified the GZT by adding 4 kinds of Chinese herbs, including *Rhizoma atractylodis macrocephalae*, *Rhizoma curcumae*, *Hedyotis diffusa*, and *Scutellaria barbata*. *Rhizoma atractylodis macrocephalae* improves chronic atrophic gastritis in rats [12]. Sesquiterpenes from the *Rhizoma curcumae* have cytotoxicity on human GC AGS cell [13]. Additionally, *Hedyotis diffusa* is a well-known herb that is frequently applied to GC treatment. Its polysaccharide, iridoids, and sfingolipids compositions have significant antitumor effects on the GC [14, 15]. The study has shown that the ethanol extract of *Scutellaria barbata* induces apoptosis through Caspase-, MAPK-, and ROS-dependent pathways, and it exerts chemotherapeutic effects in human gastric adenocarcinoma cells [16]. Therefore, it is of great interest to explore the therapeutic effects and potential molecular mechanisms of modified GZT in GC. The human gut is home to trillions of microorganisms, primarily bacteria, forming a complex network that plays a crucial role in regulating the immune system, metabolizing cytotoxins, and maintaining overall metabolism [17]. Intestinal flora is associated with various tumors, such as esophageal cancer [18], colorectal cancer [19], and GC [20]. Previous study shows that *H. pylori* infection is the primary risk factor for GC [21]. With the advanced sequencing techniques, it has been found that other microbiota and microbial community alterations also contribute to cancer in the stomach. For example, *H. suis*, *H. felis*, *Helicobacter salomonis*, and *Helicobacter bizzozeronii*, which are non-*H. pylori*, are associated with GC [22]. In terms of abundance and diversity, the intestinal flora changed significantly during the progression from gastritis to GC [23]. TCM can regulate the gut microbiota. After treatment with TCM, the number and activity of probiotics in the intestinal flora increase, the pathogens are suppressed, and the intestinal microenvironment is maintained in balance [24]. Notably, Huangqi Jianzhong and Xiangsha Liujunzi decoctions have demonstrated their effectiveness in treating *H. pylori* infection [25]. However, whether GZT affects the development of GC through the regulation of intestinal flora has been rarely reported.

Chronic gastritis undergoes a process of intestinal metaplasia and gastric intraepithelial neoplasia, eventually transforming into GC [26]. Pyroptosis, also known as inflammatory programmed cell death, promotes the maturation of interleukin-18 (IL-18) and IL-1β by activating caspases proteins (caspase-1/-11/-4/-5) [27]. Pyroptosis is a complex process that can be classified into two distinct pathways, namely the caspase-1-dependent classical pathway and the caspase-4/5/11-dependent non-classical pathway. Caspase 1, by cleaving GSDMD, forms 10–21 nm pores in the host cell membrane from which cellular inflammatory factors and danger-associated molecular patterns are released, leading to the regulation of gut microbiota homeostasis [28, 29]. CagA, a key factor in *H. pylori* pathogenesis, promotes migration and invasion of GC cells through activation of the NLRP3 inflammasome [30]. *Callicarpa nudiflora*, a component of TCM, decreases the release of lactate dehydrogenase (LDH), tumor necrosis factor-alpha (TNF-α), IL-1β, IL-6, and IL-8, as well as the expression of caspase-1, and NLRP3, to inhibit *H. pylori*-induced pyroptosis and protect gastric epithelial cells [31]. However, whether the GZT affects pyroptosis by regulating intestinal flora in GC remains unknown.

In this study, we examined the effects of low-dose, and high-dose of modified GZT treatment on GC tumor growth, proliferation, metastasis, invasion, and caspase-1-dependent pyroptosis through in vivo experiments. The impact of modified GZT on intestinal flora was investigated by 16S rRNA sequencing. Finally, we explored whether the modified GZT inhibited the development of GC by regulating intestinal flora. This study investigated the correlation between the modified GZT and GC progression, intestinal flora and pyroptosis, offering a theoretical foundation for the clinical treatment of GC using modified GZT.

## Materials and methods

### Modified GZT preparation

Based on the original GZT formula and through a series of animal experiments, the optimal formula for the treatment of GC was ultimately established. The modified GZT contains including *Peach kernel* (9 g), *Safflower* (9 g), *Angelica sinensis* (9 g), *Chuanxiong* (6 g), *Red peony* (6 g), *Peony bark* (6 g), *YanhuSuo* (3 g), *Wulingzhi* (6 g), *Wuyao* (6 g), *Fructus Aurantii* (4.5 g), *Xiangfu* (4.5 g), *Licorice* (9 g), *Rhizoma atractylodis macrocephalae* (12 g), *Rhizoma curcumae* (9 g), *Hedyotis diffusa* (15 g), and *Scutellaria barbata* (15 g). Free Decoction pellets of the modified GZT were obtained from Jiangyin Tianjiang Pharmaceutical Co., Ltd (Wuxi City, Jiangsu Province, China). The herbs were mixed and pulverized. Then, they were extracted using ultrasonication three times (60 min each time) with 70% alcohol and 30% pure water. The supernatant was collected, and alcohol was removed using rotary evaporation. The remaining extract was then freeze-dried to obtain a powdered form.

### HPLC-mass spectrometry (HPLC–MS/MS) analysis of modified GZT

LC–MS grade methanol and acetonitrile for extraction or analysis were purchased from CNW Technologies. LC–MS grade formic acid was obtained from Sigma-Aldrich (St. Louis, Missouri, USA). L-2-Chlorophenylalanine (Purity ≥ 98%) was purchased from Shanghai Hengbai Biotechnology Co., Ltd. (Shanghai, China). Free decoction pellets of the modified GZT (50 mg) were accurately weighed and added to 1000 μL of extraction solution (methanol: water = 4:1, with an internal standard concentration of 10 μg/mL). The mixture was subjected to ultrasonication in an ice-water bath for 5 min. The extracted samples were allowed to stand at −40 ℃ for 1 h and then centrifuged at 12,000 rpm for 15 min. The resulting supernatant was filtered through a 0.22 μm micropore filter membrane, and stored at −80 ℃. The samples were analyzed in both negative and positive modes using an ACQUITY UPLC BEH C18 column (1.7 μm 2.1*100 mm; Waters Corporation, Ireland) under the Ultimate 3000 (Thermo Fisher Scientific, Waltham, Massachusetts, USA). The injection volume was 5 μL. Mobile phase A was 0.1% (V/V) formic acid/water and mobile phase B was 0.1% (V/V) formic acid/acetonitrile. The linear gradient elution processes for samples were presented in Additional file 2: Table S1. An Q Exactive mass spectrometer coupled with an Xcalibur software (Thermo Fisher Scientific) was employed to obtain the MS and MS/MS data based on the IDA acquisition mode. During each acquisition cycle, the mass range was from 100 to 1500, and the top three of every cycle were screened and the corresponding MS/MS data were further acquired. The detailed parameters are as follows: Sheath gas flow rate: 30 Arb; Aux gas flow rate: Capillary temperature 350 °C; Full millisecond resolution: 70,000; MS/MS resolution Collision energy: 15/30/45 (NCE mode); Spray voltage: 5.5 kV (positive) or −4.0 kV (negative).

### Cell culture

Mouse GC cell line (MFC), acquired from the icellbioscience Biotechnology Co., Ltd (Shanghai, China), were cultured using 1640 complete medium (89% of 1640 basal medium, 10% FBS, 1% of double antibody). The cells were grown in an incubator under conditions of 37 °C with 5% CO_2_.

### Tumor xenograft animal models construction and drug treatment

Total 18 BALB/c male mice (5 ~ 6 weeks old, weighing 18–20 g) were obtained from the SPF (Beijing) Biotechnology Co., Ltd. The mice were provided with sufficient food and water and kept in a pathogen-free environment. They were housed in cages under standard laboratory conditions, with temperatures ranging from 22 to 24 °C, relative humidity levels maintained at 55–60%, and a 12-h light/dark cycle. The mice were randomly allocated to three groups: the Model group, the Lower Dose group, and the Higher Dose group. After 1 week of adaptive feeding, tumor xenograft models were constructed by subcutaneously injecting 100 μL of 5 × 10^6^/mL MFC cell suspension into the mice's inguinal area. After the injection, the needle was slowly withdrawn and a clear bulge was seen at the injection site. All experimental protocols were approved by the Animal Care and Use Committee of Southern Medical University (Guangzhou, China).

Free decoction pellets of the modified GZT were melted with double-distilled water. The Lower Dose and the Higher Dose groups were gavaged daily with 0.2 mL and 0.4 mL of the modified GZT, respectively, while the Model group received an oral administration of an equivalent amount of sterile water. Every 7 days, the caliper was used to measure the width (W) and length (L) of the tumor to monitor its growth. The tumor volume (V) = (W^2^ × L)/2. After 28 days, the mice were euthanized and the tumor tissues were aseptically extracted. The tumors were evaluated for volume and weight, and images of the tumor tissues were captured for documentation.

### Hematoxylin–Eosin staining (HE)

The mouse tumor tissues were fixed using 4% paraformaldehyde and subsequently dehydrated with xylene. The paraffin-embedded tumor tissues were sectioned into slices, and subsequently subjected to deparaffinization using xylene and dehydration with graded ethanol. The sections were stained with hematoxylin (Sigma, St. Louis, MO, USA) for 10 min, differentiated briefly in 0.7% hydrochloric acid in ethanol for, and then stained with alcoholic eosin (Sigma) for 30 s. After cleaning with xylene, the sections were sealed with neutral gum. The stained sections were visualized using a light microscope (Olympus, Tokyo, Japan).

### Immunohistochemistry (IHC)

The mouse tumor tissue was fixed using 4% paraformaldehyde, then embedded in paraffin and sliced into 4-μm thick sections. The paraffin was removed with a citrate buffer solution (pH 6.0) and the sections were soaked in 3% H_2_O_2_ for 25 min. Following blocking with goat serum for 1 h, the sections were incubated overnight at 4 °C with Ki67 antibody (1:500, Abcam, Cambridge, UK), GSDMD antibody (1:500, Abcam), and GSDME antibody (1:500, Abcam). On the following day, the sections were stained with HRP-labeled secondary antibody for 30 min. Then the sections were restained with hematoxylin, dehydrated in gradient ethanol, cleared with xylene, and sealed with neutral gum. Immunohistochemical images were obtained under a light microscope (Olympus), and cell proliferation rates were calculated using ImageJ software.

### Western blotting

Total proteins were isolated from mice tumor tissues using RIPA lysate containing protease and phosphatase inhibitors (Solarbio, Beijing, China). The total protein concentration was measured using the BCA protein assay kit (Solarbio). Then 10%-sodium dodecyl sulfate polyacrylamide gels were prepared, and 30 μg of protein was loaded to each well for electrophoresis. Proteins were transferred from the gels onto the PVDF membranes (Roche, Basel, Switzerland) and blocked with 5% non-fat milk for 1 h. The PVDF membranes were then incubated overnight at 4 °C with primary antibodies, followed by incubation with secondary antibodies at room temperature for 1 h. Protein bands were visualized using enhanced chemiluminescence reagents (Amersham, Little Chalfont, UK). The primary antibodies were: anti-CD147 (1:2000, Abcam), anti-VEGF (1:2000, Abcam), anti-MMP-9 (1:2000, Abcam), anti-NLRP3(1:2000, Abcam), anti-ASC (1:2000, Abcam), anti-caspase-1(1:2000, Abcam), and anti-β-actin (1:2000, Abcam).

### Enzyme-linked immunosorbent assay (ELISA)

The concentrations of TNF-α, IL-1β, IL-18, and LDH in mouse serum were quantified using ELISA kits (Esebio, Shanghai, China). All ELISA procedures were carried out in accordance with the instructions provided by the manufacturer. The absorbance was recorded at 450 nm using a microplate reader (DALB, Shanghai, China).

### Fecal microbiota transplantation (FMT)

The FMT detection method was based on the description of a previous study [32]. Briefly, feces from donor mice in the Model and Higher Dose group were collected and stored at -80℃. The feces were resuspended in sterile phosphate-buffered saline at a concentration of 100 mg/mL, centrifuged at 1000 × g for 3 min, and the supernatant was collected for transplant material.

After adaptation for 1 week, BALB/c male mice were treated with a combination of antibiotics (1 g/L ampicillin, neomycin, and metronidazole, and 0.5 g/L vancomycin) in the drinking water for 4 weeks. At the same time, 100 μL MFC cells (5 × 10^6^) were injected into the inguinal area of all mice, which were divided into two groups: Model (FTM) and Higher dose (FTM) with feces collected from donor mice in the Model and Higher Dose group, respectively. The mice were then orally fed 200 μL of the fecal suspension once a day for consecutive 28 days. Mice for the control group were treated with normal water and inguinal injection with 0.9% saline solution.

### Extracting genomic DNA from mice feces

The extraction of genomic DNA from fecal samples of mice was performed using the E.Z.N.A. soil DNA Kit (Omega Bio-Tek, GA, USA) according to the manufacturer's protocol. Subsequently, the NanoDrop 2000 spectrophotometer (Thermo Fisher Scientific) was employed to evaluate the concentration and purity of the DNA, while the quality of the DNA was confirmed through 1% agarose gel electrophoresis.

### 16S rRNA sequencing of the intestinal flora

The ABI GeneAmp 9700 PCR Thermocycler (ABI, CA, USA) was utilized to amplify the V3-V4 hypervariable region of the bacterial 16S rRNA gene. PCR began with initial denaturation for 3 min at 95 °C, followed by 27 cycles of 30 s at 95 °C, an annealing for 30 s at 55 °C, extension for 45 s at 72 °C, and a single extension for 10 min at 72 °C. The PCR products were purified using the AxyPrep DNA Purification Kit (Axygen Biosciences, CA, USA), which were measured using 2% agarose gel electrophoresis. Subsequently, the PCR products were quantified by QuantiFluor-ST Fluorometer (Promega, Madison, WI, USA), and paired-end sequencing (2 × 300) of purified and pooled amplicon libraries was performed on the Illumina MiSeq platform (Illumina, San Diego, USA) based on the standard protocol of Majorbio Bio-Pharm Technology Co., Ltd., (Shanghai, China).

### Bioinformatics analysis of the intestinal flora

Sequences were classified into Operational Taxonomic Units (OTUs) at a 97% similarity level. Taxonomic information was obtained by comparing the optimized sequences with the SILVA 16S rRNA database (https://www.arb-silva.de/) for annotation. The Usearch program (version 7.0.1090) was used, based on the Ribosomal Database Project (RDP) (http://rdp.cme.msu.edu), to cluster the OTUs, allowing for annotation of taxonomic levels ranging from phylum to genus. Alpha diversity was calculated using the vegan package in the R language. Principal coordinates analysis (PCoA) was performed using the R package (http://www.R-project.org/) to show the beta-diversity of the microbiome between samples. Principal component analysis (PCA) and orthogonal partial least squares discriminant analysis (OPLS-DA) were performed using SIMCA-P software (Version 14.1, Umetrics, Umea, Sweden) to analyze the data.

### Statistics

All experiments were repeated three times, and the data were analyzed using GraphPad Prism (version 7.0) and R language (version 4.0.2). The results were displayed as means ± standard deviation (SD). Student's t-test (unpaired, two-tailed) was used to establish significant levels for comparisons between two groups. For significant differences between more than two groups, Tukey's test-corrected one-way analysis of variance (ANOVA) was performed. The Kruskal Wallis test was used to compare data that did not conform to normal distribution. The p-value less than 0.05 was considered statistically significant.

## Results

### Metabolite analysis of modified GZT

A total of 39,978 metabolites were identified in modified GZT samples by UPLC-MS/MS analysis. The metabolites in modified GZT primarily included 4-methylcatechol, ailanthone, 18β-glycyrrhetinic acid, (all-E)-6'-apo-y-caroten-6'-al, 2-(3,4-dihydroxyphenylethyl)-6-epi-elenaiate, 2,5-dihydroxybenzoic acid, 3,4-dihydroxybenzoic acid. The top 10 metabolites from negative ion model and positive ion mode were selected for Total Ion Chromatogram peak display, respectively, based on scoring values (Additional file 1: Figures S1 and S2; Additional file 2: Tables S2 and S3).

### Modified GZT inhibits GC tumor development

**Fig. 1:**
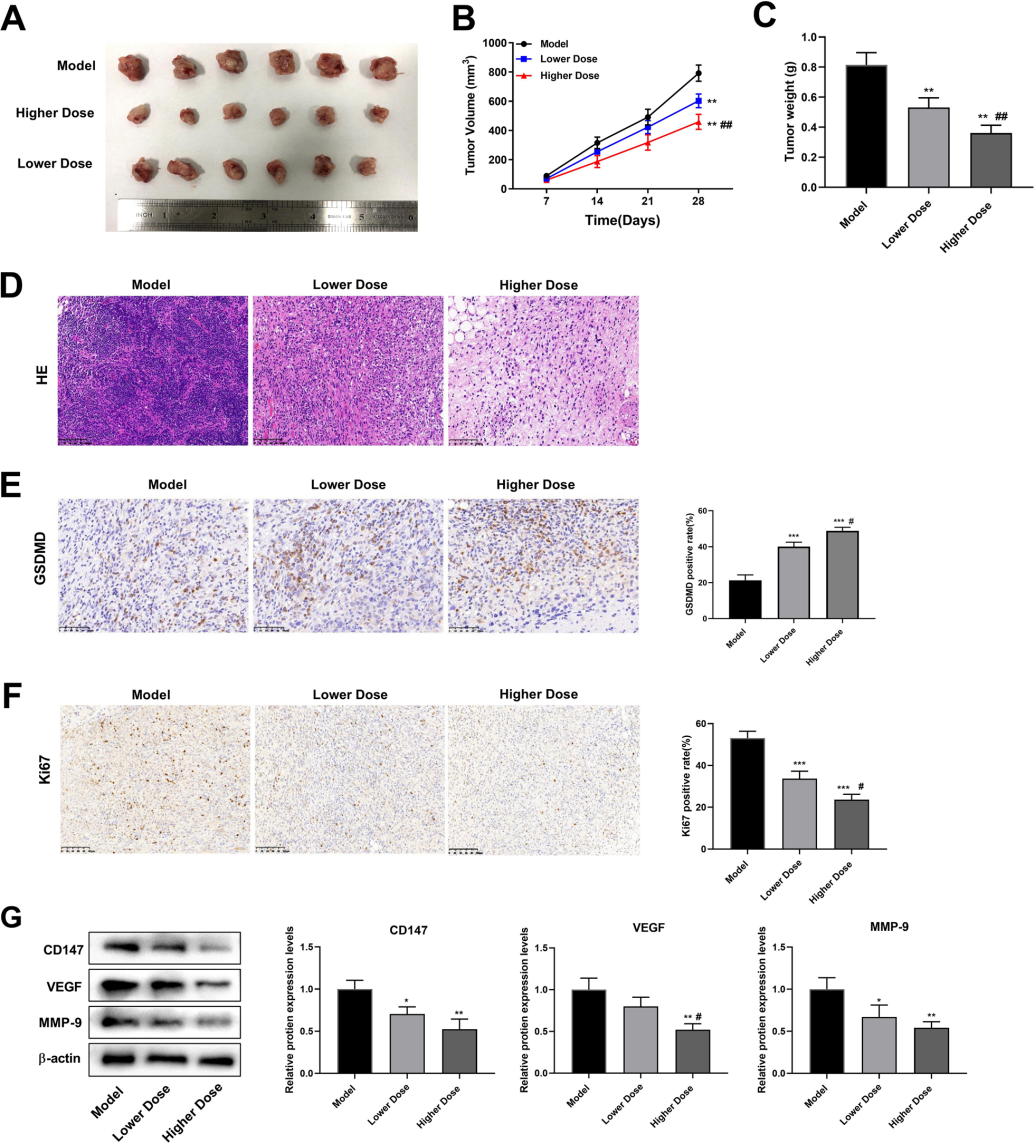
The modified GZT treatment inhibits tumor tumorigenicity and proliferation in vivo. **A** Representative xenograft tumors in the Model, Lower Dose and Higher Dose groups. **B** Tumor volume growth curve. **C** Tumor weight growth curve. **D** HE staining to observe histopathological changes (200 × , scale bar: 100 μm). **E** IHC staining was employed to measure GSDMD expression (magnification: 200 × , scale bar: 50 μm). **F** IHC staining for Ki67 (200 × , scale bar: 100 μm). **G** The modified GZT treatment inhibits the expression of tumor progress-related proteins. ^**^P < 0.01 *vs.* Model group; ^##^P < 0.01 *vs.* Lower Dose group

To explore the in vivo functionality of the modified GZT, we constructed tumor xenograft models. The results revealed that in comparison to the Model group, the tumor volume and weight were considerably reduced in the Lower Dose and Higher Dose groups. Additionally, there were significant differences between the Lower Dose group and the Higher Dose group (Fig. 1A–C). HE staining demonstrated that the tumor tissues of mice in the Model group exhibited obvious inflammatory cell infiltration, tight cell arrangement, and condensed and solidified nuclei. In contrast, the cell arrangement was slightly loosened in the Lower Dose group. The Higher Dose group demonstrated a significant change in cellular arrangement, which was characterized by a clear cellular hierarchy and a significant reduction in inflammatory cells (Fig. 1D). Subsequently, we explored the impact of modified GZT on pyroptosis. IHC showed that GSDMD expression was significantly increased in both the Lower Dose and High Dose groups compared with the Model group. Notably, GSDMD expression was significantly higher in the High Dose group than in the Lower Dose group (Fig. 1E). Furthermore, modified GZT inhibited the proliferation of GC tumor cells as evidenced by the reduction of Ki67 expression levels (Fig. 1F). In addition, studies have shown that CD147, VEGF, and MMP-9 significantly promote the invasion and metastasis of GC [33–35]. Our findings indicated that modified GZT treatment substantially reduced protein expression levels of CD147, VEGF, and MMP-9 (p < 0.05, Fig. 1G). Taken together, modified GZT inhibited GC tumor growth, proliferation, metastasis, invasion and promoted pyroptosis.

### Modified GZT promotes caspase-1-dependent pyroptosis

**Fig. 2:**
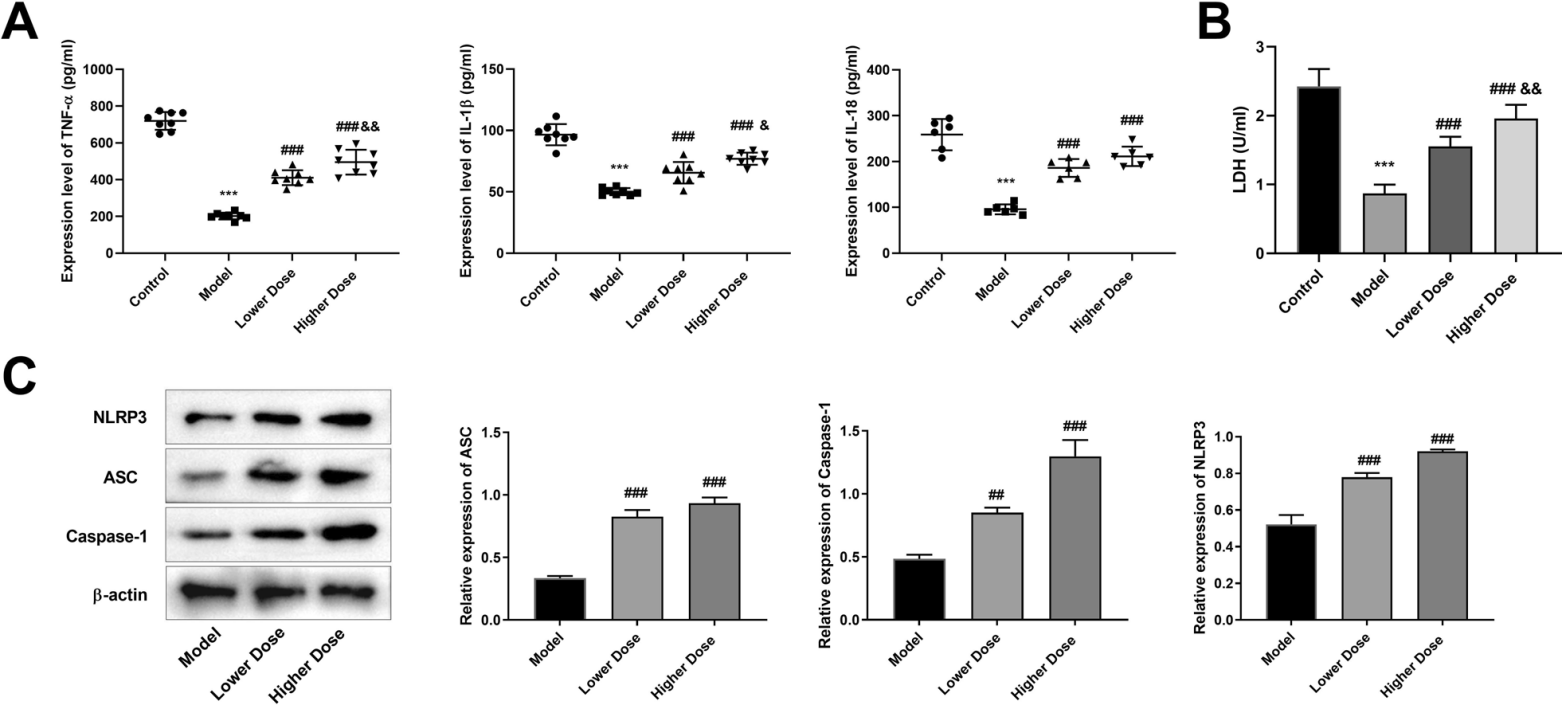
The modified GZT treatment affects the expression of inflammation-related factors and pyroptosis-related proteins. **A**–**B** ELISA was used to detect the expression levels of cytokines, including TNF-α, IL-1β and IL-18 and B LDH release. **C** The expression levels of NLRP3, ASC, and caspase-1 was detected by Western blot. ^**^P < 0.01 *vs*. Control group. ^*#*^P < 0.05; ^*##*^P < 0.01 *vs*. Model group, ^&^P < 0.05, ^&&^P < 0.01 *vs.* Lower Dose group

The molecular mechanism of modified GZT induced pyroptosis was subsequently investigated. We found that GC model had a marked decrease in TNF-α, IL-1β, and IL-18 levels comparison with the Control group, which was reversed by low and high dose modified GZT treatment. Notably, there was a significant difference between the low-dose treated group and the high-dose treated group (Fig. 2A). The LDH, a marker of plasma membrane pore formation indicative of pyroptosis, was also measured. The modified GZT treatment significantly elevated LDH levels (Fig. 2B). Western blot results indicated modified GZT both at low and high doses increased the expression of NLRP3, ASC, and caspase-1 (Fig. 2C).

### Modified GZT affects intestinal flora

**Fig. 3:**
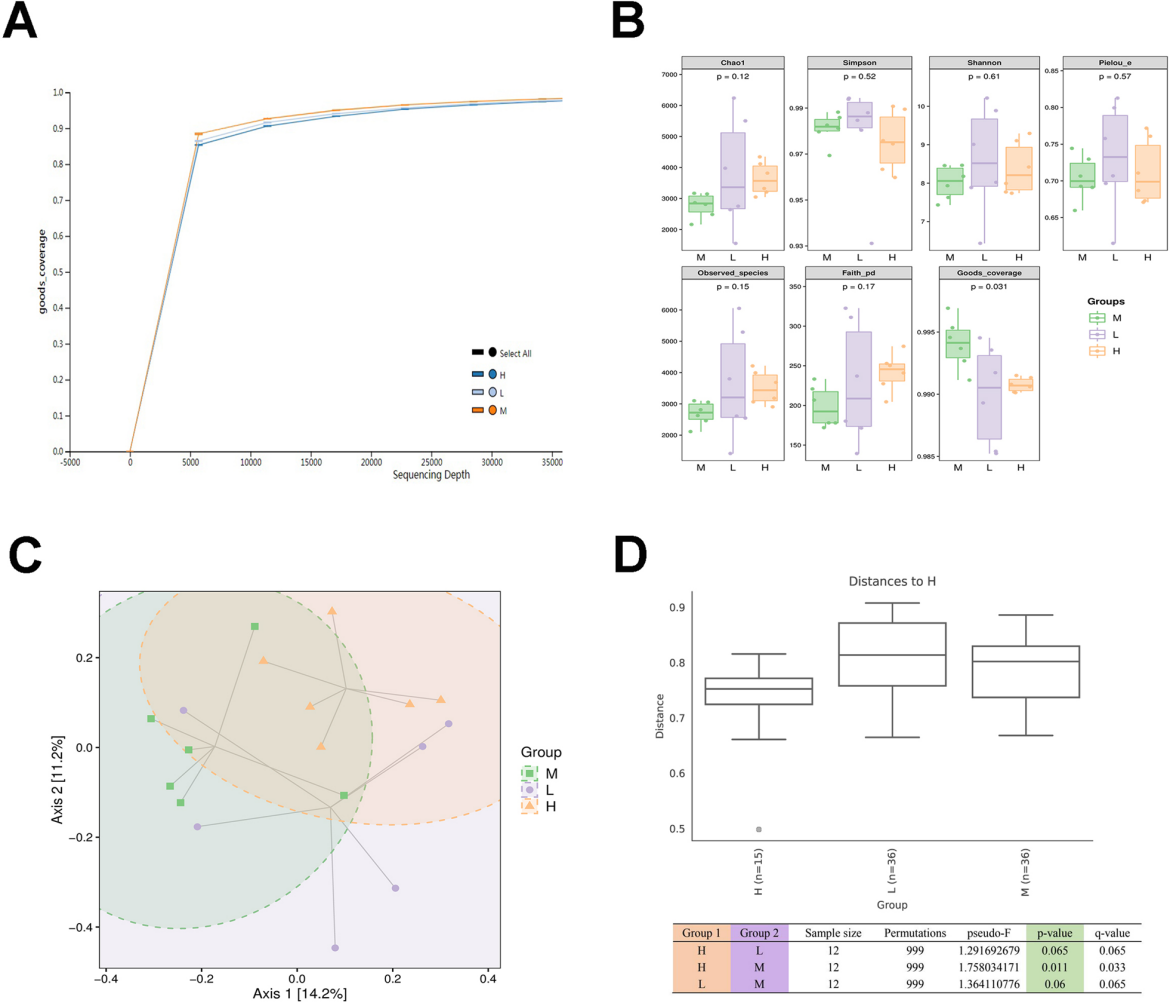
16S rRNA sequencing was used to detect the effect of the modified GZT treatment in GC on the abundance and diversity of intestinal flora. **A** The sparse curves of alpha diversity analysis. **B** Grouped box line plots of Alpha diversity indices. **C** Two-dimensional ranking plots of samples for PCoA analysis in terms of Beta diversity. **D** Box line plots of multiple group comparisons based on distance matrix and significance analysis of differences between three groups

To investigate the role of modified GZT treatment on the intestinal flora in mice with GC, we conducted high-throughput sequencing analysis of the 16S rRNA. The results of the rarefaction curves by alpha diversity analysis indicated that all samples had good sampling coverage, demonstrating that the sequencing depth was sufficient for analyzing the fecal microbiota (Fig. 3A). The indexes of Goods-coverage were significantly different in the M (Model), L (Lower Dose), and H (Higher Dose) groups (Fig. 3B). The PcoA results revealed that the differences in the number and distribution of species between the M and the H groups were significantly different (Fig. 3C). In the analysis of differences between groups, groups M and H showed significant differences in the overall, while there was no statistical difference between the M and L, and L and H groups (Fig. 3D).

**Fig. 4:**
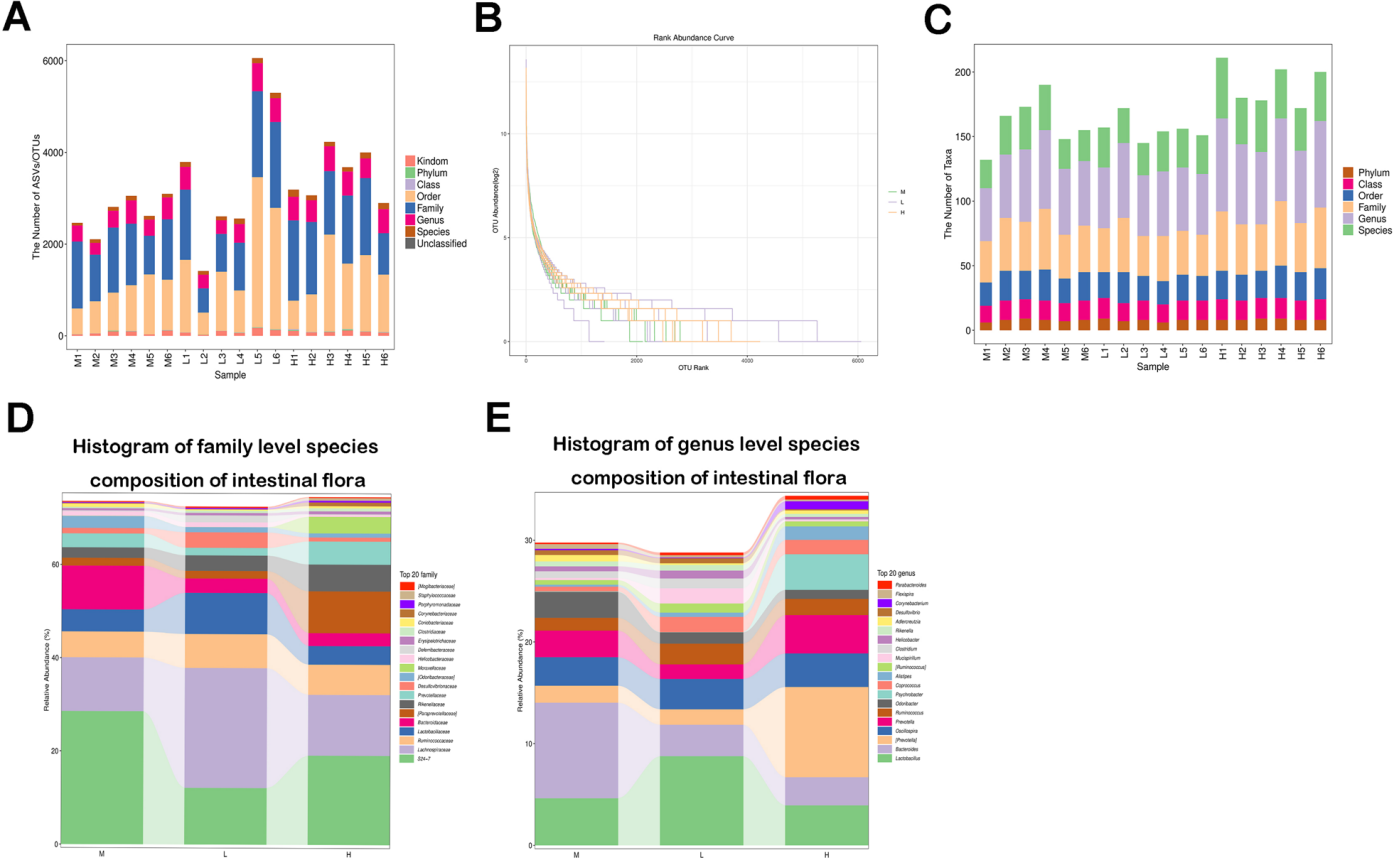
The modified GZT treatment affects the landscape of the intestinal flora in mice with GC. **A** Statistical graph of OTU classification and taxonomic status identification results. The horizontal coordinates indicate the sample names, and the vertical coordinates indicate the number of OTUs in each sample that can be classified to each taxonomic level of phylum, order, family, genus and species. **B** Relative abundance of OTUs in individual samples in the Model group (M), Lower Dose group (L), and the Higher Dose group (H). The horizontal coordinate represents the ordinal number of ASV/OTU in order of abundance, and the vertical coordinate represents the log2 logarithmic value of the abundance (mean) of each ASV/OTU. **C** Statistical plots of the number of microbial taxonomic units in different groups. The vertical coordinate represents the number of taxonomic units at each of the six levels: phylum, order, family, genus, and species. **D**–**E** Top 20 names at the family and genus level in the Model (M), the Lower (L), and the Higher Dose (H) groups

We then evaluated the composition of the gut microbiota and found that the abundance and diversity of microbial species were significantly higher in the L and H groups compared to the M group (Fig. 4A, B). Additionally, the number of microbial taxas in the H group was significantly greater than in the M group (Fig. 4C). Next, we listed the top 20 families and genera in the three groups (Fig. 4D, E). At the family level, *Bacteroidaceae* exhibited a significantly high abundance in the M group, while *Paraprevotellaceae* showed a relatively high abundance in the H group. At the genus level, the abundance of *Bacteroides* was relatively high in the M group, whereas the abundance of *Prevotella* and *Psychrobacter* were significantly high in the H group.

**Fig. 5:**
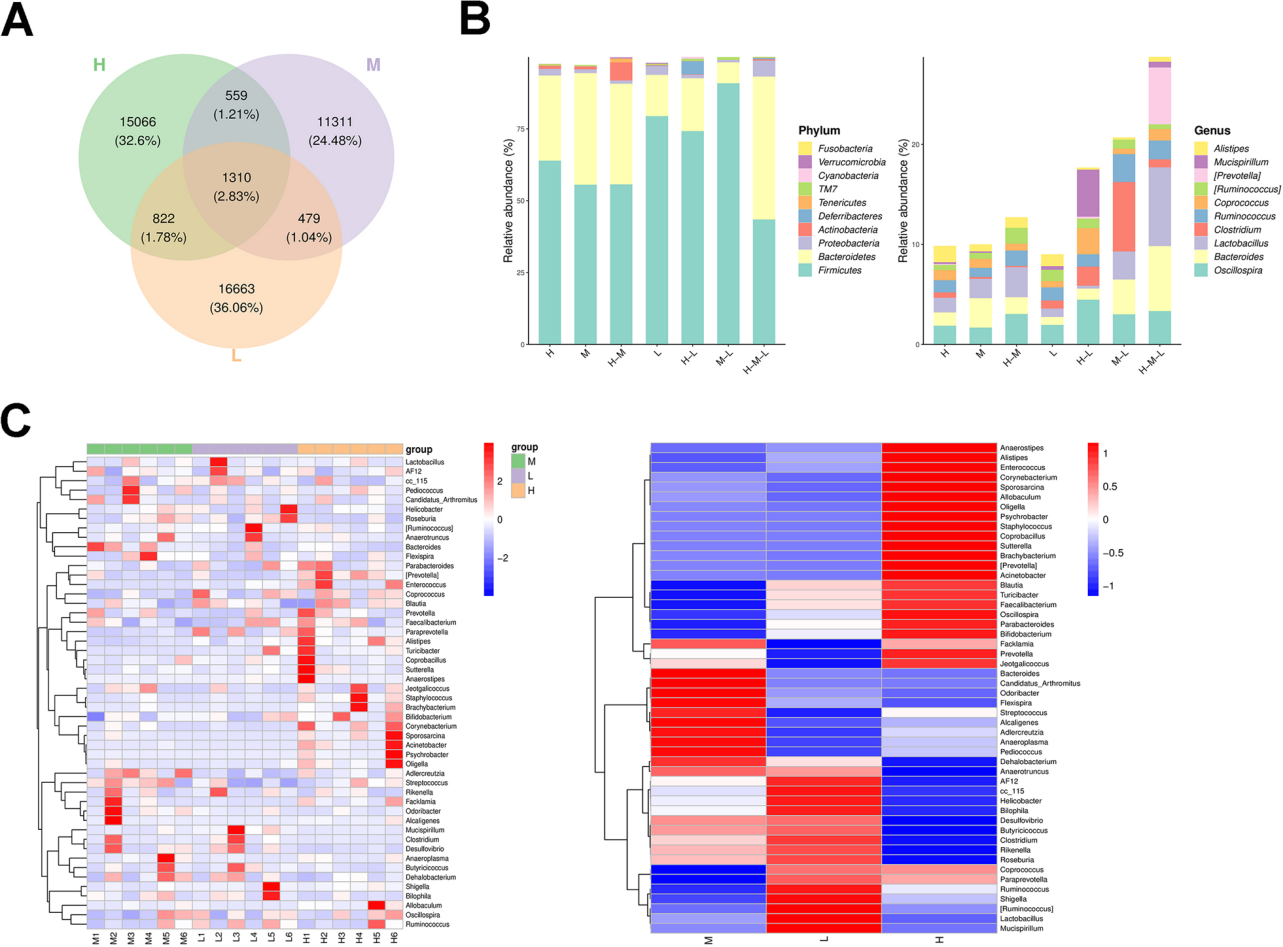
The modified GZT treatment alters the community of intestinal flora in mice with GC. **A** Venn diagram showed the overlapping OTUs in different groups. **B** Average relative abundance of gut microbiota at the phylum and genus level, respectivly, in different regions of the Venn diagram. **C** Differences in gut microbiota in the Model, Lower dose, and Higher dose group at the genus level were demonstrated by heatmaps

The Venn diagram showed that a total of 1869 OTUs overlapping between the M and H groups, accounting for 6.33%, while the H group had 15888 unique OTUs, accounting for 53.77% (Fig. 5A). At the phylum level, the *phyla Firmicutes*, *Bacteroidetes*, and *Proteobacteria* were relatively abundant (Fig. 5B). With high-dose modified GZT treatment, the abundance of *Bacteroidetes*, *Proteobacteria*, and *Tenericutes* increased, and the abundance of *Firmicutes* decreased. At the genus level, the relative abundance of *Bacteroides*, *Lactobacillus*, and *Oscillospira* ranked in the top three. In the H group, the abundance of *Bacteroides* decreased compared to the M group, while the abundance of *Coprococcus* and *Psychrobacter* increased (Fig. 5B). The heatmap was generated for the top 50 genera based on the mean abundance. The high-dose modified GZT treatment reduced the abundance of *Desulfovibrio*, *Helicobacter*, and *Facklamia*, while increased the abundance of *Alistipes*, *Sutterella*, *Paraprevotella*, and *Psychrobacter* (Fig. 5C).

By analyzing the iconic species, notable differences in ASV/out were found between the M group and the H group (Additional file 1: Figure S3A). The abundance of *Desulfovibrionaceae* and *Desulfovibrio* was significantly higher in the M group compared to the H group (Additional file 1: Figure S3B). The analysis of marker species showed significant differences in the abundance of species between the M group and the H group (Additional file 1: Figure S3C). The taxonomic hierarchy, from phylum to genus, in each group of samples was shown in Additional file 1: Figure S1D. At the order, family, and genus levels, the marker species in the modified GZT high-dose treatment group were *Turicibacterales*, *Turicibacteraceae*, and *Turicibacter*.

The PCA method was used to study the degree of dispersion of the differential species. The results demonstrated a distinct trend of separation between the M and H groups, with the ratio of the difference in species abundance composition to the total difference for all samples at 43.3% (Additional file 1: Figure S4A). At the same time, the results of OPLS-DA indicated that the abundance composition of the differential species between the M and H groups exhibited a separation trend with only a few overlapping individual parts and a high overall separation, indicating a better classification effect (Additional file 1: Figure S4B).

### Modified GZT inhibits the GC development by regulating intestinal flora

**Fig. 6:**
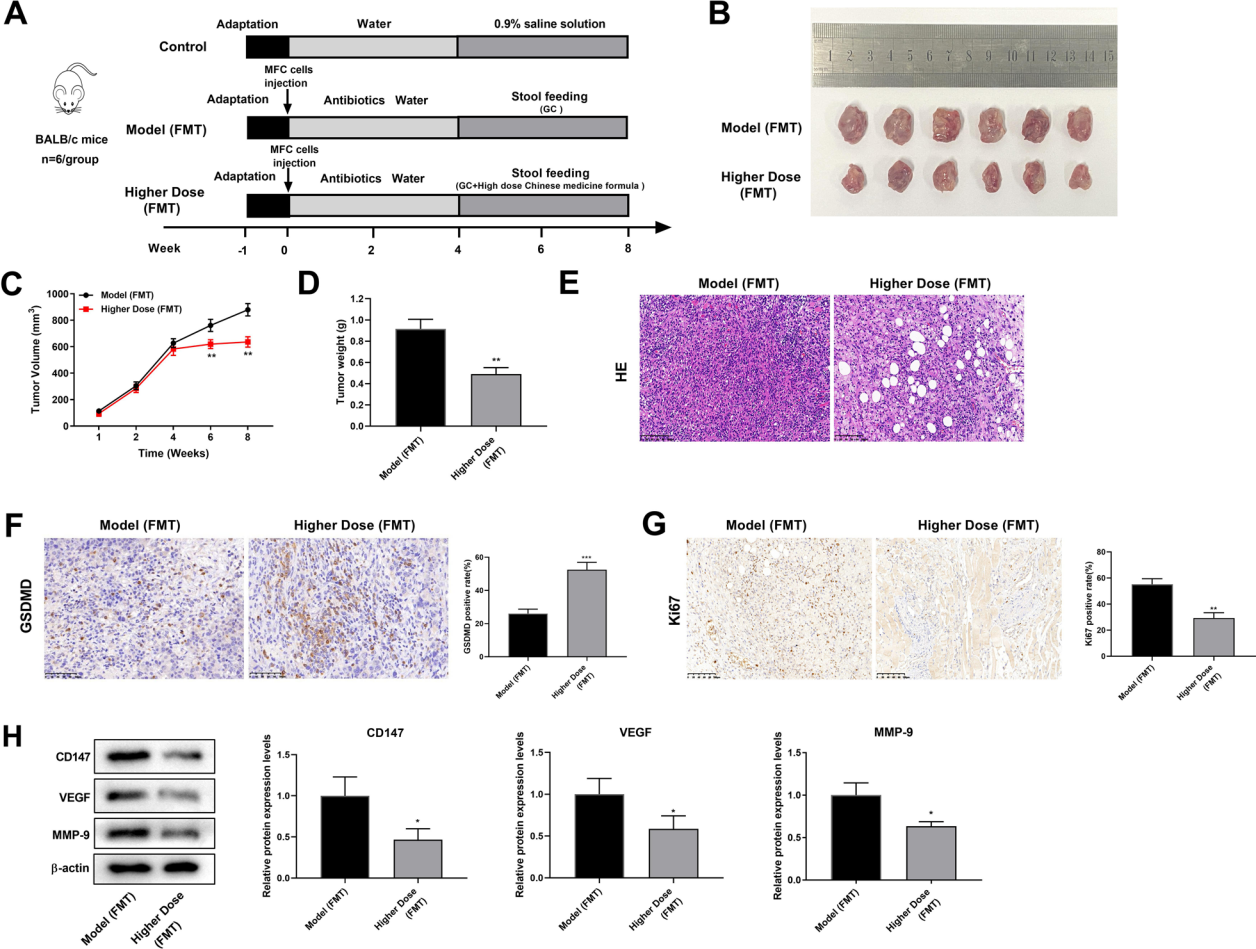
The modified GZT treatment inhibits tumor growth in vivo by regulating intestinal flora. **A** Schematic diagram of fecal gavage experiment in BALB/c mice. **B** Photographs of tumors in mice. **C** Tumor volume growth curve. **D** Tumor weight change curve. **E** Histopathological changes observed by HE staining (200 × , scale bar: 100 μm); **F** The expression level of GSDMD was assessed by IHC (magnification: 200 × , scale bar: 50 μm). **G** Ki67 immunohistochemical staining was used to detect cell proliferation (200 × , scale bar: 100 μm). **H** Western blot was used to detect the protein expression levels of CD147, VEGF and MMP-9 in the Model (FMT) and the Higher Dose (FMT) group. ^*^P < 0.05; ^**^P < 0.01 *vs*. Model group

The *Paraprevotella*, *Psychrobacter*, *Trematoda*, *Alistipes*, *Coprococcus*, *Oscillospira*, and *Sutterella* are not the predominant bacterial community in GC. Therefore, the study opted for a more comprehensive analysis by using the entire fecal microbiota for the FMT experiment, which provided insights into how GZT treatment affected the gut microbiota and its potential impact on gastric cancer in mice. Fecal microorganisms from donor mice were extracted and transferred to recipient mice to evaluate the impact of FMT on GC progression in mice (Fig. 6A). The results indicated that a noteworthy decrease was observed in both the tumor volume and weight in the high-dose of the modified GZT (FMT) group compared to the Model (FMT) group (Fig. 6B–D). Moreover, there was a significant reduction in immune cell infiltration and inflammatory cells in the Higher Dose (FMT) group compared to the Model (FMT) group (Fig. 6E). IHC assay results showed a significant increase in GSDMD expression in the Higher Dose (FMT) group compared with the Model (FMT) group (Fig. 6F). Furthermore, modified GZT (FMT) decreased the number of Ki67-positive cells (Fig. 6G). The expression levels of metastasis and invasion-associated proteins CD147, VEGF, and MMP-9 were significantly lower in the Higher Dose (FMT) group than in the Model (FMT) group (Fig. 6H).

### Modified GZT promotes pyroptosis by regulating gut microbiota

**Fig. 7:**
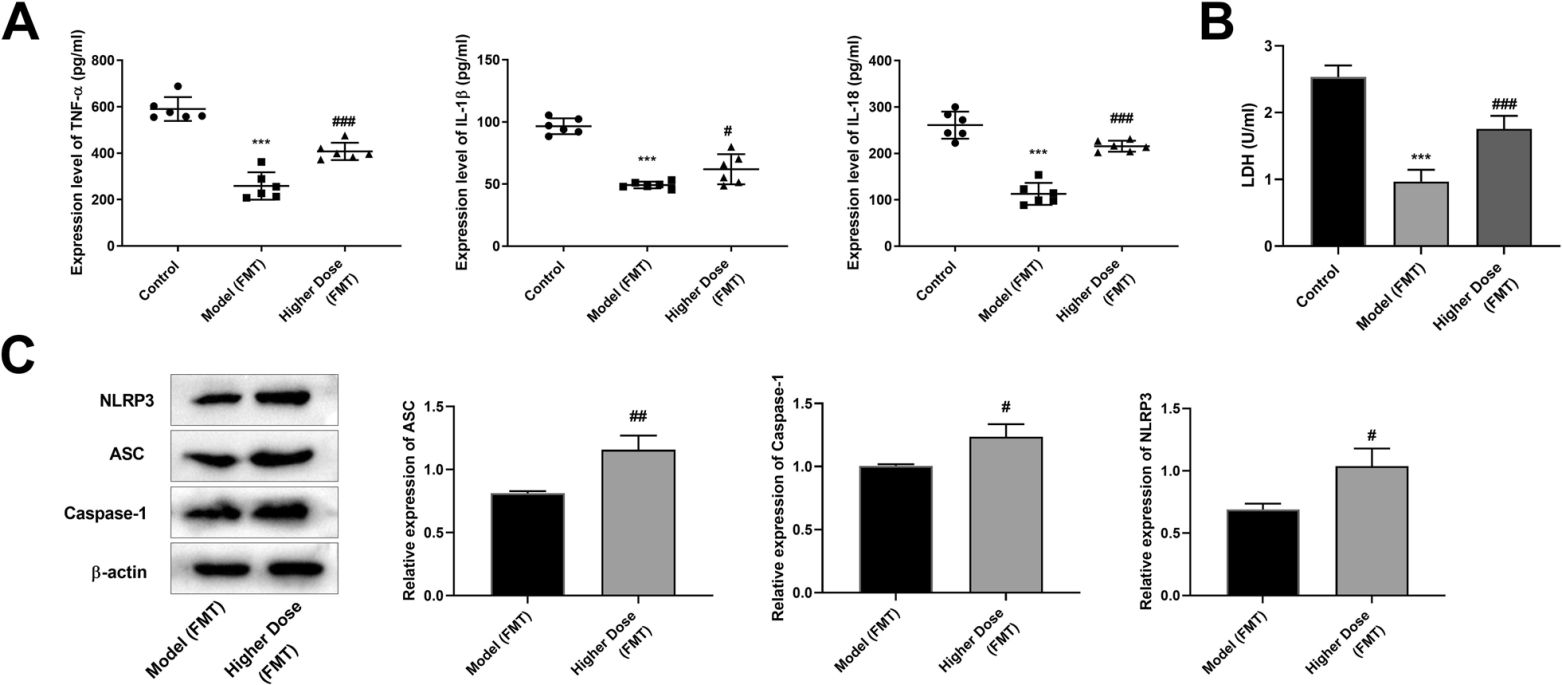
The modified GZT inhibits pyroptosis by regulating gut microbiota. **A**–**B** ELISA assay was used to detect the expression levels of TNF-α, IL-1β, IL-18 and LDH. **C** Western blot was used to detect the protein expression of NLRP3, ASC, and caspase-1. ^*^P < 0.05; ^**^P < 0.01 *vs*. Control group; ^##^P < 0.01 *vs*. Model group

Next, we used FMT to detect whether gut microbiota altered by modified GZT had therapeutic benefits for GC. Fecal microbiota from Model or higher dose modified GZT-treated mice were transplanted into GC mice recipients. ELISA analysis showed that the levels of TNF-α, IL-1β, IL-18, and LDH levels were significantly lower in the Model (FMT) group compared to the Control group. In contrast, the levels of TNF-α, IL-1β, IL-18, and LDH levels in the Higher Dose (FMT) group were significantly higher than that in the Model (FMT) group (Fig. 7A, B). Furthermore, the expression of NLRP3, ASC, and caspase-1 proteins were increased in the Higher Dose (FMT) group than that in the Model (FMT) group (Fig. 7C).

## Discussion

GC is a malignancy that originates from the inner lining of the stomach with a high morbidity and mortality rate [36]. *H. pylori* infection, diet, family history, and alcohol consumption are all high risk factors for GC [37], of which *H. pylori* infection is responsible for approximately 70% of all GC cases worldwide [38]. Advances in molecular biology and sequencing technologies have enabled researchers to study the gut microbiome in greater depth and breadth in GC, not just *H. Pylori*. 16S rRNA sequencing has proven to be a powerful tool for exploring the diversity and composition of microbial communities. In the gut microbiome, 16S rRNA sequencing has been employed to study the effects of diet, drug therapy, and disease on microbial diversity and composition [39]. In the current study, we demonstrated that modified GZT inhibited the growth, proliferation, metastasis, invasion, and promoted pyroptosis of GC. Through 16S rRNA sequencing analysis, we detected alterations in the gut microbiota following modified GZT treatment. This study demonstrates that modified GZT effectively suppresses GC progression by modulating the gut microbiota.

First, we analyzed the metabolites of modified GZT by HPLC–MS/MS. The results showed that the metabolites of modified GZT mainly included dihydroxybenzoic acid, 4-methylcatechol, ailanthone, and 18β-glycyrrhetinic acid. In GC 3,4-dihydroxybenzoic acid promotes cell apoptosis through JNK/p38 MAPK signaling activation and exerts anti-tumor effects [40]. The 18β-glycyrrhetinic acid exhibits properties with potential benefits, including antitumor, anti-inflammatory, and antiviral effects [41–43]. Furthermore, 18β-glycyrrhetinic acid impedes the development and advancement of GC by downregulating COX-2 expression and suppressing Wnt-1 expression through miR-149–3 upregulation [44]. It inhibits the migration and invasion of GC cells through the ROS/PKC-α/ERK pathway [45]. 4-methylcatechol induces apoptosis via the intrinsic mitochondrial pathway and demonstrates cytotoxic effects on metastatic malignant melanoma cells [46]. Ailanthone possesses anti-inflammatory, anti-malarial, and anti-tumor effects. It can inhibit the progression of multiple tumors, including acute myeloid leukemia, lung, breast, melanoma, and gastric cancer [47]. These findings all suggest that modified GZT may have an important role in inhibiting tumor development.

Then we explored the effects of modified GZT on GC. The results revealed that modified GZT significantly inhibited tumor growth in a dose-dependent manner. The Ki-67 marker is a recognized parameter for measuring proliferation in malignant tumors [48]. IHC results showed that modified GZT reduced the number of Ki67-positive cells, inhibiting the proliferation of GC tumor tissue cells. CD147, which is highly expressed on the extracellular surface of numerous types of tumor tissue, potentially promotes tumor metastasis through its regulation of cellular substrate and adhesion mechanisms [49]. The role of CD147 in promoting tumor invasiveness has been recently confirmed in various human malignancies, including GC [33]. Angiogenesis plays a crucial role in the metastasis of solid tumors, with VEGF standing out as the most potent mediator of angiogenesis [50]. MMP-9, a member of the matrix metalloproteinase family, is involved in GC metastasis and invasion [51]. In the present study, we found that both low and high doses of modified GZT suppressed the expression of CD147, VEGF, and MMP-9, implying that modified GZT inhibited the metastasis and invasion of GC tumors. In addition, IHC staining demonstrated that modified GZT significantly increased the expression level of GSDMD. GSDMD is a widely investigated pore-forming protein in pyroptosis, and it is a substrate for activated caspase-1/4/5/11 [52]. Further investigation showed that modified GZT greatly promoted the protein expression levels of NLRP3, ASC and caspase-1, and the concentrations of TNF-α, IL-1β, IL-10 and LDH. Subsequent FMT showed that modified GZT inhibited the development of GC by modulating the gut microbiota.

Composition and species abundance of intestinal flora are significantly altered in GC. Studies have shown that as the progression from gastritis to intestinal metaplasia and GC occurs, there is a gradual decline in both diversity and abundance of gut microbiota [23, 53]. In GC, the abundance of *Fusobacterium*, *Leptotrichia*, *Veillonella*, *Campylobacter* and *Haemophilus* is notably elevated [54]. In comparison to patients with intestinal metaplasia and gastritis, those with GC exhibit a significantly higher abundance of *Lactobacillus*, *Clostridium*, and *Lachnospiraceae* [55]. The fecal microbiota promotes inflammation, affects cell proliferation and immune regulation, and disrupts DNA integrity, ultimately contributing to carcinogenesis [56]. The most common Helicobacter species is *H. Pylori*. The mechanism through which *H. pylori* infection induces GC mainly involves chronic gastritis, releasing gastrin and histamine, damaging DNA, and activating the proliferation of gastric epithelial cells through multiple pathways such as the induction of the PI3K/Akt pathway [57]. The increased abundance of *Lachnospiracea* in GC may be related to inflammatory regulation [58]. In our study, we found that after high-dose modified GZT treatment, the abundances of many gut flora species and the community composition were significantly changed. At the genus level, the abundance of several genera including *Bacteroides*, *Desulfovibrio*, *Helicobacter*, and *Facklamia* decreased, while the abundance of *Paraprevotella*, *Psychrobacter*, *Trematoda*, *Alistipes*, *Coprococcus*, *Oscillospira*, and *Sutterella* increased.

Numerous studies have shown that TCMF can regulate intestinal flora and affect the development of intestinal cancer. A previous study has found that the intestinal flora diversity, microbial composition, and abundance in patients with precancerous lesions of GC are altered after treatment with Weifuchun capsules [59]. Quxie Capsules elevate the abundance of *Actinobacteria* and *Lachnospiraceae*, while decrease the abundance of *Bacteroides*, *Bacteroidetes*, *Escherichia Shigella*, and *Gammaproteobacteria*, which may help protect against colorectal cancer tumors and enhance immunity [60]. Curcumin, a compound generated by the roots of the Curcuma longa plant, may have anti-carcinogenic qualities via preserving the diversity of gut bacteria [61]. Antibiotics are extensively employed in the realm of Western medicine for the management of a broad spectrum of ailments [62]. Nevertheless, their usage is concomitant with an alteration in the composition and a reduction in the diversity of the human microbiota [63, 64]. Investigation has revealed a progressive increase in the risk of GC associated with the cumulative number of penicillin courses administered [65]. Antibiotic administration not only influences the resistome of the subject to whom it is given, but also the whole population owing to selection for resistance to its function [66]. Prior research has demonstrated that the use of antibiotics in conjunction with prebiotics, probiotics, and synbiotics assists the gut microbiota in resisting *H. pylori* infections and lowers the proportion of drug-resistant bacteria [67]. Therefore, we hypothesize that modified GZT combined with antibiotic therapy plays a role in preserving gut microbiota homeostasis and decreasing drug resistance.

Inflammasomes in the gastrointestinal tract are associated with intestinal flora homeostasis and infection [68]. Studies have shown that TCM can reduce *H. pylori*-induced gastritis by decreasing the expression of IL-8, TNF-α, IL-6, iNOS, and IFN-γ [69]. These findings suggest that there is an important correlation between TCM and intestinal flora, inflammation, and pyroptosis. In this study, we found that high dose modified GZT facilitated the expression proinflammatory factors TNF-α, IL-1β, IL-18, and caspase-1-dependent pyroptosis by modulating intestinal flora. Caspase-1 activates primarily the pro-inflammatory factors IL-1β and IL-18 [70]. Moreover, studies have shown that some pathogen-associated molecular patterns, carried by bacteria, are delivered to the host cytoplasm where they activate inflammasomes [68]. Thus, we speculate that modified GZT promotes caspase-1-dependent pyroptosis by inhibiting harmful bacteria, which in turn enhances the release of proinflammatory factors.

However, there are also limitations in this investigation. Firstly, we relied on previous studies for GZT ingredients by HPLC analysis and did not conduct HPLC analysis of modified GZT in this study due to scientific limitations. Secondly, for the assessment of pyroptosis, we determined the expression levels of GSDMD, inflammatory factors, and pyroptosis-related proteins using IHC assay. However, morphological analysis was not performed through electron microscopy due to scientific limitations. Additionally, in the evaluation of modified GZT effect on the metastasis and invasion of GC, we solely assessed the protein expression levels of CD147, VEGF, and MMP-9, which will be further validated by in vitro experiments in our subsequent exploration.

## Conclusion

In this study, we found that the modified GZT inhibited the growth, proliferation, metastasis, and invasion of GC by regulating the intestinal flora. Furthermore, modified GZT also promoted pyroptosis of GC. The current investigation revealed a novel therapeutic strategy and theoretical foundation for utilizing TCMF in the treatment of GC.

### Supplementary Information

**Additional file 1: Figure S1.** Total Ion Chromatogram peak of the top 10 metabolites from negative ion model. **Figure S2.** Total Ion Chromatogram peak of the top 10 metabolites from positive ion model. **Figure S3.** Analysis of marker species of intestinal flora. A. MetagenomeSeq test results in the Model and the Higher Dose group. B. The log2 values of the top five ASV/OUT and ASV/OTU multiples that were significantly up- and down-regulated. Positive values represent upregulation in M group compared with H or L group. C. Histogram of distribution of LDA values for significantly different species. D. Taxonomic branching diagram showing taxonomic hierarchy relationships from phylum to genus in each taxon sample. **Figure S4.** PCA and OPLS-DA analysis of differential species. A. Two-dimensional sorting diagram of samples for PCA analysis. B. Sorting diagram of samples for OPLS-DA discriminant analysis.

**Additional file 2: Table S1.** The gradient elution process of modified GZT. **Table S2.** The top 10 metabolites of modified GZT from negative ion model. **Table S3.** The top 10 metabolites of modified GZT from positive ion model.

## Data Availability

The datasets used and/or analysed during the current study are available from the corresponding author on reasonable request.

## Abbreviations

TCMF: Traditional Chinese medicine formula
GC: Gastric cancer
GZT: Gexia-Zhuyu Tang
FMT: Fecal microbiota transplantation
HE: Hematoxylin–eosin
IHC: Immunohistochemical
ELISA: Enzyme-linked immunosorbent assay
HPLC: High-performance liquid chromatography
AQPs: Aquaporins
TNF-α: Tumor necrosis factor-alpha
IL-1β: Interleukin-1 beta
IL-18: Interleukin-18
LDH: Lactate dehydrogenase
OTUs: Operational taxonomic units
RDP: Ribosomal database project
PCoA: Principal coordinates analysis
PCA: Principal component analysis
OPLS-DA: Orthogonal partial least squares discriminant analysis
SD: Means ± standard deviation
ANOVA: Analysis of variance
