# Supplementary material for: Modified Gexia-Zhuyu Tang inhibits gastric cancer progression by restoring gut microbiota and regulating pyroptosis
Source: Cancer Cell Int. 2024 Jan 9;24:21. doi: 10.1186/s12935-024-03215-6 (PMC10775600; doi:10.1186/s12935-024-03215-6)

Additional Figure 1 Total Ion Chromatogram peak of the top 10 metabolites from negative ion model.


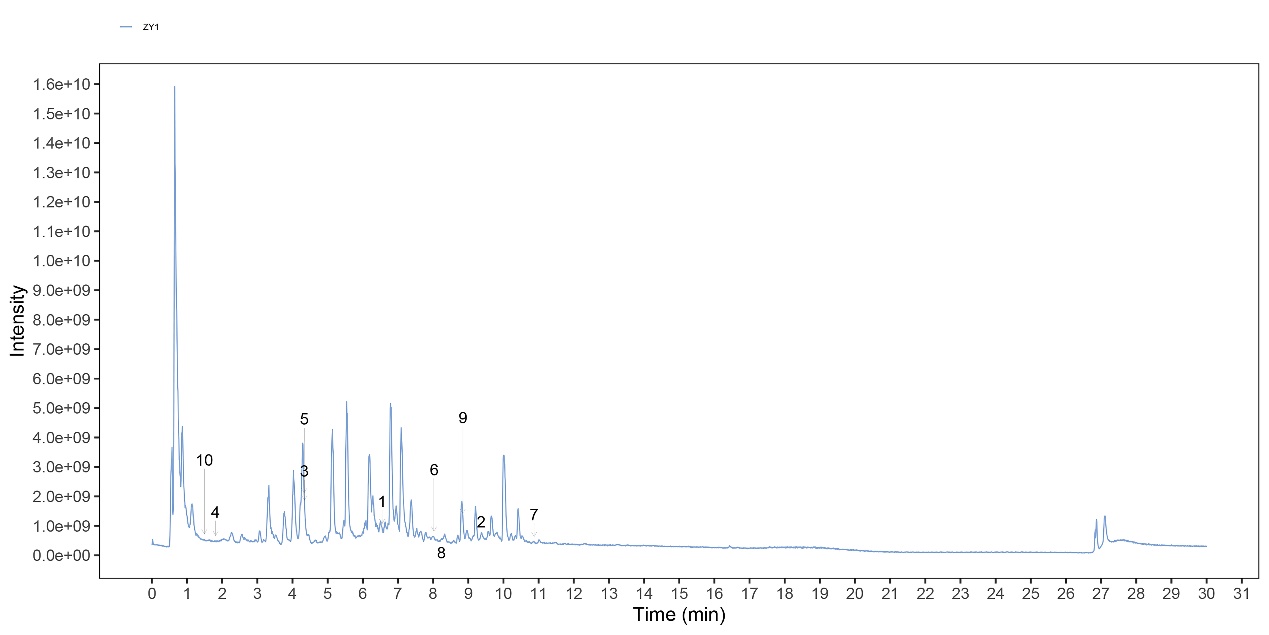


Additional Figure 2 Total Ion Chromatogram peak of the top 10 metabolites from positive ion model.


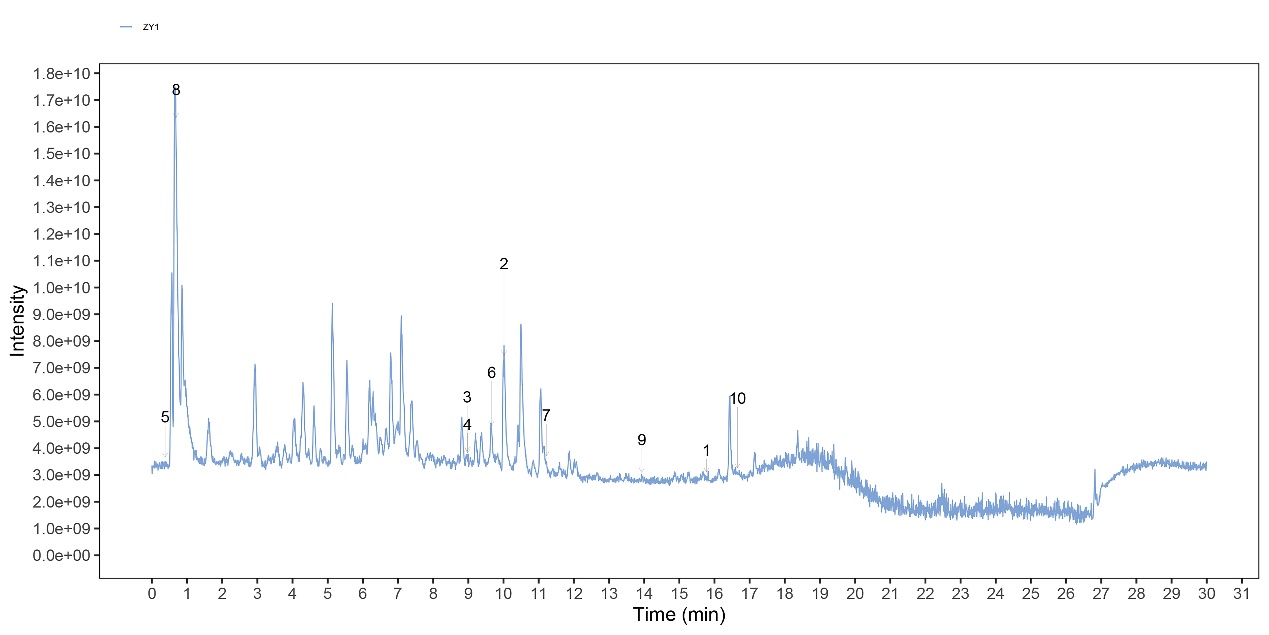


Additional Figure 3 Analysis of marker species of intestinal flora. A. MetagenomeSeq test results in the Model and the Higher Dose group. B. The log2 values of the top five ASV/OUT and ASV/OTU multiples that were significantly up- and down-regulated. Positive values represent upregulation in M group compared with H or L group. C. Histogram of distribution of LDA values for significantly different species. D. Taxonomic branching diagram showing taxonomic hierarchy relationships from phylum to genus in each taxon sample.
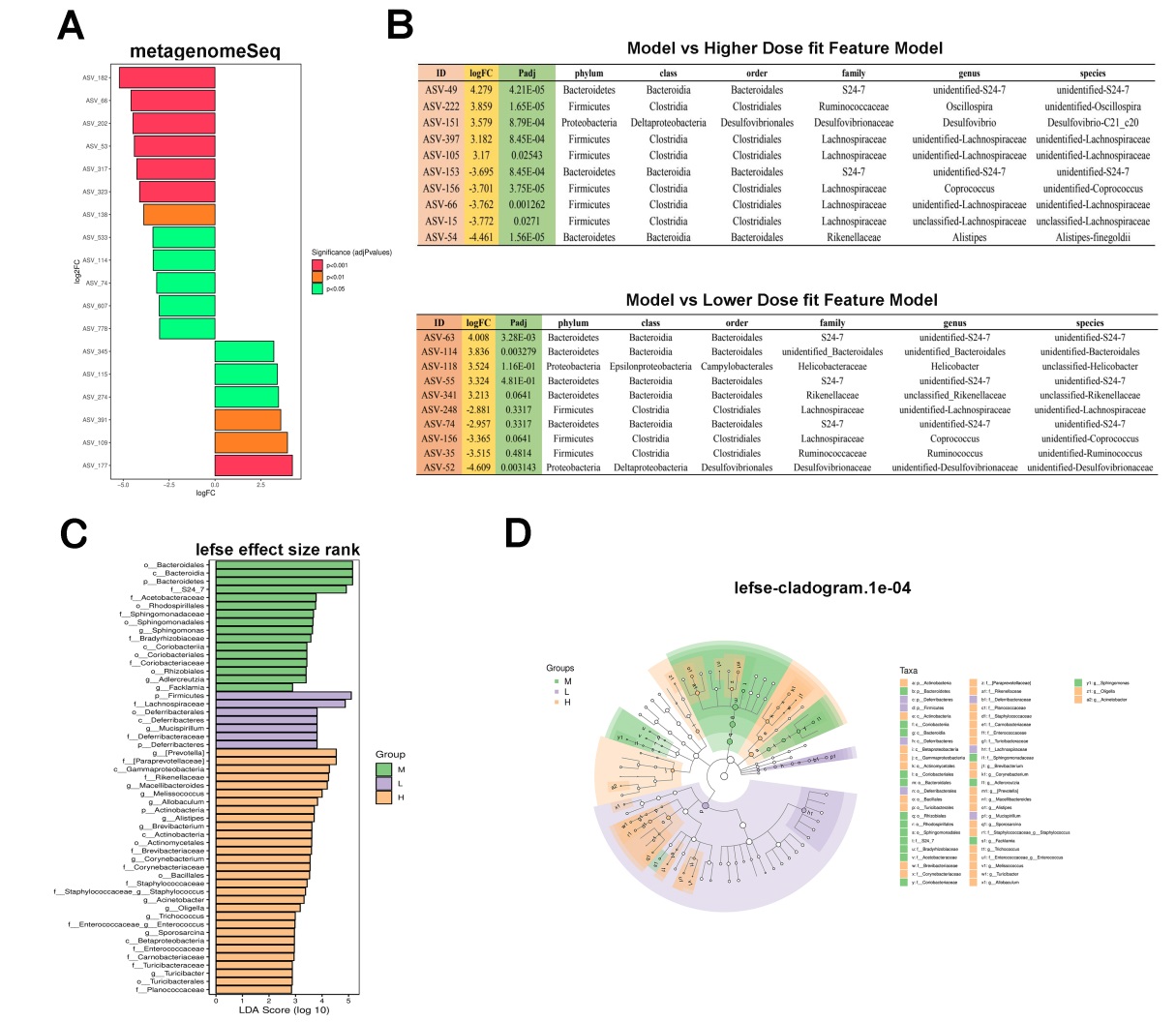


Additional Figure 4. PCA and OPLS-DA analysis of differential species. A. Two-dimensional sorting diagram of samples for PCA analysis. B. Sorting diagram of samples for OPLS-DA discriminant analysis.


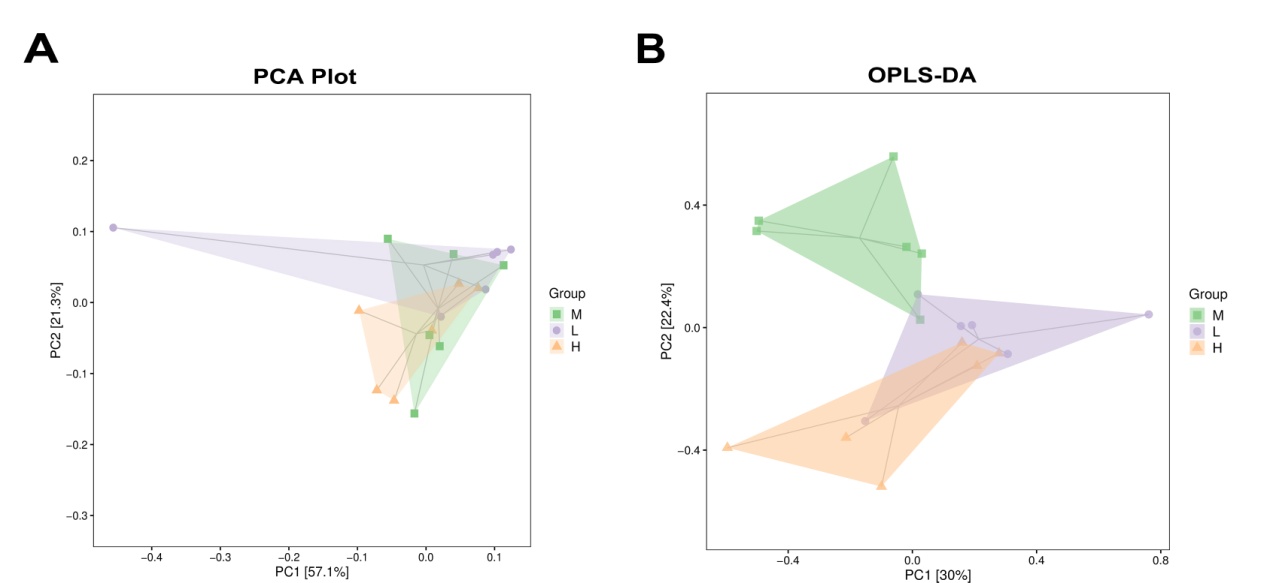

Supplement: Supplementary file 1 — Additional file 1: Figure S1. Total Ion Chromatogram peak of the top 10 metabolites from negative ion model. Figure S2. Total Ion Chromatogram peak of the top 10 metabolites from positive ion model. Figure S3. Analysis of marker species of intestinal flora. A. MetagenomeSeq test results in the Model and the Higher Dose group. B. The log2 values of the top five ASV/OUT and ASV/OTU multiples that were significantly up- and down-regulated. Positive values represent upregulation in M group compared with H or L group. C. Histogram of distribution of LDA values for significantly different species. D. Taxonomic branching diagram showing taxonomic hierarchy relationships from phylum to genus in each taxon sample. Figure S4. PCA and OPLS-DA analysis of differential species. A. Two-dimensional sorting diagram of samples for PCA analysis. B. Sorting diagram of samples for OPLS-DA discriminant analysis. [file 12935_2024_3215_MOESM1_ESM.docx]
