# Supplementary material for: Modified Gexia-Zhuyu Tang inhibits gastric cancer progression by restoring gut microbiota and regulating pyroptosis
Source: Cancer Cell Int. 2024 Jan 9;24:21. doi: 10.1186/s12935-024-03215-6 (PMC10775600; doi:10.1186/s12935-024-03215-6)
Supplement: Supplementary file 2 — Additional file 2: Table S1. The gradient elution process of modified GZT. Table S2. The top 10 metabolites of modified GZT from negative ion model. Table S3. The top 10 metabolites of modified GZT from positive ion model. [file 12935_2024_3215_MOESM2_ESM.docx]

Additional table 1 The gradient elution process of modified GZT

| Time (min) | Flow rates (μL/min) | A% water | B% acetonitrile |
| --- | --- | --- | --- |
| 0 | 400 | 95 | 5 |
| 3.5 | 400 | 85 | 15 |
| 6 | 400 | 70 | 30 |
| 6.5 | 400 | 70 | 30 |
| 12 | 400 | 30 | 70 |
| 12.5 | 400 | 30 | 70 |
| 18 | 400 | 0 | 100 |
| 22 | 400 | 0 | 100 |
| 25 | 400 | 0 | 100 |
| 26 | 400 | 95 | 5 |
| 30 | 400 | 95 | 5 |

Additional table 2 The top 10 metabolites of modified GZT from negative ion model

| NameEN | Formula | mzmed | rtmed | MS2 |  |  |  |  |
| --- | --- | --- | --- | --- | --- | --- | --- | --- |
| (2S,3R,5S,9R)-2,3-dihydroxy-14-(hydroxymethyl)-5,9-dimethyl-14-{[(2S,3R,4S,5S,6R)-3,4,5-trihydroxy-6-(hydroxymethyl)oxan-2-yl]oxy}tetracyclo[11.2.1.0¹,¹⁰.0⁴,⁹]hexadecane-5-carboxylic acid | C26H42O11 | 529.264486 | 392.863 | 529.260899;530.266749;354.09455;85.027313;113.022961 | | | | |
| [(1S,3R,5R,6S,8S)-3-{[(2S,3R,4S,5S,6R)-6-[(benzoyloxy)methyl]-3,4,5-trihydroxyoxan-2-yl]oxy}-6-hydroxy-8-methyl-9,10-dioxatetracyclo[4.3.1.0²,⁵.0³,⁸]decan-2-yl]methyl benzoate | C30H32O12 | 629.186605 | 562.169 | 121.02828;630.637772;605.942762;553.171441;210.210453 | | | | |
| 2,5-Dihydroxybenzoic acid | C7H6O4 | 153.017594 | 260.443 | 109.027132;153.017682;153.866619;110.03026;108.020291 | | | | |
| 3,4-Dihydroxybenzoic acid | C7H6O4 | 153.017588 | 108.835 | 153.017534;153.866724;124.03818;82.028205;122.892011 | | | | |
| 4-Methylcatechol | C7H8O2 | 123.04329 | 259.919 | 123.043331;124.039868;95.048548;121.028271;108.030939 | | | | |
| 5,9-dihydroxy-5,7,7-trimethyl-4,5a,6,8,8a,9-hexahydro-1H-azuleno[5,6-c]furan-3-one | C15H22O4 | 265.143143 | 481.482 | 265.1439;266.14631;219.846212;57.033017;90.183829 | | | | |
| 7-hydroxy-2-phenyl-4H-chromen-4-one | C15H10O3 | 237.054639 | 652.507 | 237.053891;238.057441;209.059216;236.046316;208.050547 | | | | |
| Ailanthone | C20H24O7 | 375.143595 | 494.164 | 375.142212;376.150201;342.109472;298.119731;59.012086 | | | | |
| Anthraquinone base + 2O, MeOH | C15H10O5 | 269.044075 | 530.657 | 269.044154;270.047316;117.033186;151.001987;149.022354 | | | | |
| Benzene-1,2,4-triol | C6H6O3 | 125.022799 | 89.538 | 125.022756;125.872172;69.032937;92.658302;96.958576 | | | | |

Additional table 3 The top 10 metabolites of modified GZT from positive ion model

| Name | Formula | mzmed | rtmed | MS2 |  |  |  |  |
| --- | --- | --- | --- | --- | --- | --- | --- | --- |
| (all-E)-6'-Apo-y-caroten-6'-al | C32H42O | 443.335175 | 946.3495 | 443.336984;444.339846;290.543717;85.313967;75.50296 | | | | |
| 18 beta-Glycyrrhetintic Acid | C30H46O4 | 471.34723 | 600.897 | 471.345131;472.347706;189.162584;135.116864;95.085808 | | | | |
| 2-(3,4-Dihydroxyphenylethyl)-6-epi-elenaiate | C19H22O8 | 379.139366 | 537.971 | 379.137468;380.111277;229.123619;233.119128;155.650742 | | | | |
| 3-(1,1-Dimethylallyl)herniarin | C15H16O3 | 245.117789 | 539.033 | 229.123164;247.133001;183.11838;211.112128;139.039064 | | | | |
| 4-Hydroxy-1H-indole-3-acetonitrile | C10H8N2O | 173.071388 | 22.6393 | 146.059853;174.053974;128.049204;173.071294;149.941067 | | | | |
| 4,4,8,10,14-pentamethyl-17-(4,5,6-trihydroxy-6-methylheptan-2-yl)-2,5,6,7,9,15-hexahydro-1H-cyclopenta[a]phenanthrene-3,16-dione | C30H46O5 | 487.342563 | 579.773 | 487.337738;488.349558;119.086014;54.149145;189.16279 | | | | |
| 7-hydroxy-1,4a-dimethyl-9-oxo-7-propan-2-yl-2,3,4,4b,5,6,10,10a-octahydrophenanthrene-1-carboxylic acid | C20H30O4 | 335.220082 | 673.214 | 335.217631;336.121609;224.65389;105.444916;214.972726 | | | | |
| Aurasperone D | C31H24O10 | 557.149346 | 41.1607 | 557.152658;558.151744;61.905597;205.470556;147.612444 | | | | |
| Chaulmoogric Acid | C18H32O2 | 298.274726 | 835.982 | 81.070319;299.25554;71.08651;95.085813;298.275964 | | | | |
| Citranaxanthin | C33H44O | 457.350962 | 999.271 | 457.348391;460.041521;217.153632;107.839769;82.109687 | | | | |
